# Systematic Review of the Quality of Stereolithographic Three-Dimensionally Printed Materials for Provisional Dental Restorations

**DOI:** 10.3390/ma18030721

**Published:** 2025-02-06

**Authors:** Alexandra Ioana Danila, Daniel Breban-Schwarzkopf, Ecaterina Daescu, Iustin Olariu, Stefania Dinu

**Affiliations:** 1 Department of Anatomy and Embriology, “Victor Babes” University of Medicine and Pharmacy Timisoara, 300041 Timisoara, Romania; alexandra.danila@umft.ro (A.I.D.); daescu.ecaterina@umft.ro (E.D.); 2 Department of Dental Medicine, Faculty of Dentistry, “Vasile Goldis” Western University of Arad, 310048 Arad, Romania; olariu.iustin@uvvg.ro; 3 Department of Pedodontics, Faculty of Dental Medicine, “Victor Babes” University of Medicine and Pharmacy Timisoara, 300041 Timisoara, Romania; dinu.stefania@umft.ro; 4 Pediatric Dentistry Research Center, Faculty of Dental Medicine, “Victor Babes” University of Medicine and Pharmacy Timisoara, 300041 Timisoara, Romania

**Keywords:** stereolithography, dental prosthesis, mechanical phenomena, systematic review

## Abstract

Background and Objectives: The use of stereolithographic (SLA) 3D printing technology in dentistry has expanded, particularly for the fabrication of provisional dental restorations. Understanding the mechanical properties and quality of SLA 3D-printed materials is essential to ensure clinical success and patient safety. This systematic review aims to critically evaluate and summarize the available evidence on the mechanical properties and quality of SLA 3D-printed materials. Methods: A comprehensive literature search was conducted in PubMed, Scopus, Embase, Cochrane, and Web of Science up to October 2024. Studies comparing the mechanical properties of SLA 3D-printed provisional restoration materials with those of milled, conventional, or other additive manufacturing methods were included. Nine studies met the inclusion criteria. Data on flexural strength, hardness, fracture resistance, surface roughness, marginal adaptation, accuracy, cement film thickness, shear bond strength, and biofilm formation were extracted and analyzed. Results: The findings from the included studies indicate that SLA 3D-printed materials exhibit varied mechanical properties. Some studies reported that SLA 3D-printed resins had significantly lower flexural strength and hardness compared to milled PMMA and bis-acrylic resins. Other studies found that SLA 3D-printed resins showed clinically acceptable marginal adaptation, surface roughness, and fracture strength comparable to those fabricated by subtractive manufacturing and conventional methods. In terms of accuracy, build orientation influenced the dimensional accuracy of SLA-printed restorations. Studies assessing cement film thickness found that SLA-printed provisional restorations had higher cement film thickness compared to other materials. Regarding repairability and fatigue resistance, limitations were observed in some SLA resins. Conclusions: The mechanical properties and quality of SLA 3D-printed materials for provisional dental restorations vary among studies. While SLA technology holds promise for efficient fabrication of provisional restorations, inconsistencies in material properties suggest a need for further research to optimize materials and printing parameters. Standardization of protocols is necessary to ensure reliable clinical performance of SLA 3D-printed provisional restorations.

## 1. Introduction

Provisional dental restorations play a crucial role in restorative dentistry, serving as interim solutions that protect prepared teeth, maintain aesthetics, and preserve occlusal relationships while the final restorations are being fabricated [1,2]. Traditionally, these restorations have been made using materials such as polymethyl methacrylate (PMMA) and bis-acrylic resins through conventional techniques like direct intraoral fabrication or indirect laboratory methods [3,4,5]. With advancements in digital dentistry, computer-aided design and computer-aided manufacturing (CAD/CAM) technologies have revolutionized the fabrication process, offering improved accuracy, efficiency, and customization [6,7].

Among the emerging technologies, additive manufacturing, commonly known as 3D printing, has gained significant attention in dentistry [8]. Stereolithography (SLA) is one of the earliest and most widely used 3D printing technologies, which constructs objects layer by layer by curing a photosensitive resin with a laser or light source [9,10]. The adoption of SLA 3D printing in dentistry allows for precise fabrication of complex geometries and detailed features, making it suitable for producing provisional restorations [11]. However, the mechanical properties and clinical performance of SLA 3D-printed materials remain a subject of ongoing research and debate.

The quality and durability of provisional restorations are critical, as they must withstand occlusal forces, resist wear, and maintain structural integrity throughout their service life [12,13]. Mechanical properties such as flexural strength, fracture resistance, and hardness are essential indicators of a material’s suitability for provisional restorations [14]. While SLA 3D printing offers advantages in customization and production efficiency, concerns have been raised regarding the mechanical performance of SLA-printed resins compared to conventional materials and fabrication methods [15].

Some research indicates that SLA-printed resins exhibit inferior flexural strength and hardness compared to milled PMMA and bis-acrylic resins, potentially limiting their clinical applicability [15]. Conversely, other studies suggest that with appropriate material selection and post-processing protocols, SLA-printed resins can achieve mechanical properties comparable to or exceeding those of traditional materials [16,17,18]. These discrepancies highlight the need for a comprehensive evaluation of the existing evidence.

In addition to mechanical properties, factors such as surface roughness and biofilm formation are important considerations for provisional restorations [19,20]. Surface roughness can affect the aesthetics, plaque accumulation, and periodontal health. Biofilm formation on restorative materials can lead to secondary caries and gingival inflammation [21]. Studies have shown that SLA-printed resins may have favorable surface properties, but data on biofilm formation and its clinical implications are limited [22].

Given the interest in SLA 3D printing for provisional dental restorations coupled with notable inconsistencies in the existing research about the quality and mechanical properties of SLA-printed materials, there is a clear need for a systematic review. This review aims to critically assess and integrate the available literature on the mechanical properties and overall quality of SLA 3D-printed materials in this context. Specifically, it will compare these materials to conventional ones and other fabrication methods, aiming to bridge the identified knowledge gap concerning their clinical viability and durability. The novelty and importance of this study lie in its potential to clarify these uncertainties, thereby informing future research directions and potentially guiding clinical practice towards more effective and reliable use of SLA printing in dental restoration. This systematic review is essential for advancing the application of SLA technology in dentistry, ensuring that both practitioners and patients benefit from the most current and robust information available.

## 2. Materials and Methods

### 2.1. Study Design and Protocol Registration

This systematic review was conducted in accordance with the Preferred Reporting Items for Systematic Reviews and Meta-Analyses (PRISMA) guidelines. The review protocol was developed to address the following research question: “What is the quality and mechanical performance of SLA 3D-printed materials used for provisional dental restorations?” This study was registered in the Open Science Framework under the registration number osf.io/r6ntm.

The PICO statement for this systematic review is as follows: Population: patients requiring provisional dental restorations; Intervention: use of SLA 3D printing for these restorations; Comparison: traditional materials like milled PMMA and bis-acrylic resins and other additive manufacturing methods; Outcome: assessment of mechanical properties such as flexural strength, hardness, fracture resistance, and other relevant characteristics. This review aims to evaluate and synthesize the mechanical properties and clinical viability of SLA 3D-printed materials compared to conventional and other 3D-printed materials.

### 2.2. Eligibility Criteria

Studies were included in this review if they met the following criteria: (1) investigated SLA 3D-printed materials used for provisional dental restorations; (2) assessed mechanical properties such as flexural strength, fracture resistance, hardness, surface roughness, or biofilm formation; (3) compared SLA-printed materials with other fabrication methods or materials; and (4) were original research articles published in peer-reviewed journals. The exclusion criteria for this systematic review were carefully delineated to focus exclusively on relevant studies. Excluded were studies that did not center on provisional dental restorations, ensuring the focus remained on the application of SLA 3D-printed materials in this specific context. Additionally, any research not employing SLA technology was omitted, as this review aims to evaluate the impacts and specifics of this particular 3D printing technology in dental restoration. This review also excluded non-original research such as review articles, case reports, and conference abstracts, which generally lack original data and detailed methodologies necessary for rigorous comparative analysis. Furthermore, studies not published in peer-reviewed journals were excluded to maintain a standard of quality and ensure the research had been subject to academic scrutiny.

### 2.3. Literature Search Strategy

A comprehensive literature search was conducted in five electronic databases, PubMed, Scopus, Embase, Cochrane, and Web of Science, up to October 2024. To ensure a thorough capture of relevant studies, our search strategy incorporated a combination of keywords and Medical Subject Headings (MeSH) terms relating to SLA 3D printing and provisional dental restorations. The primary keywords included “stereolithography”, “SLA”, “3D printing”, “additive manufacturing”, “provisional dental restorations”, “temporary crowns”, “crowns”, “mechanical properties”, “flexural strength”, and “hardness”. Boolean operators “AND” and “OR” were strategically used to combine these terms effectively, as presented in Table 1.

### 2.4. Study Selection and Data Extraction

Two independent reviewers screened the titles and abstracts of the retrieved studies to assess their eligibility based on the predefined criteria. Full-text articles of potentially relevant studies were obtained and evaluated in detail. Any disagreements between the reviewers were resolved through discussion or consultation with a third reviewer. Data were extracted using a standardized data extraction form, collecting information on study characteristics, materials and methods, mechanical properties assessed, results, and key findings. The extracted data were cross-checked for accuracy and completeness.

## 3. Results

**Figure 1:**
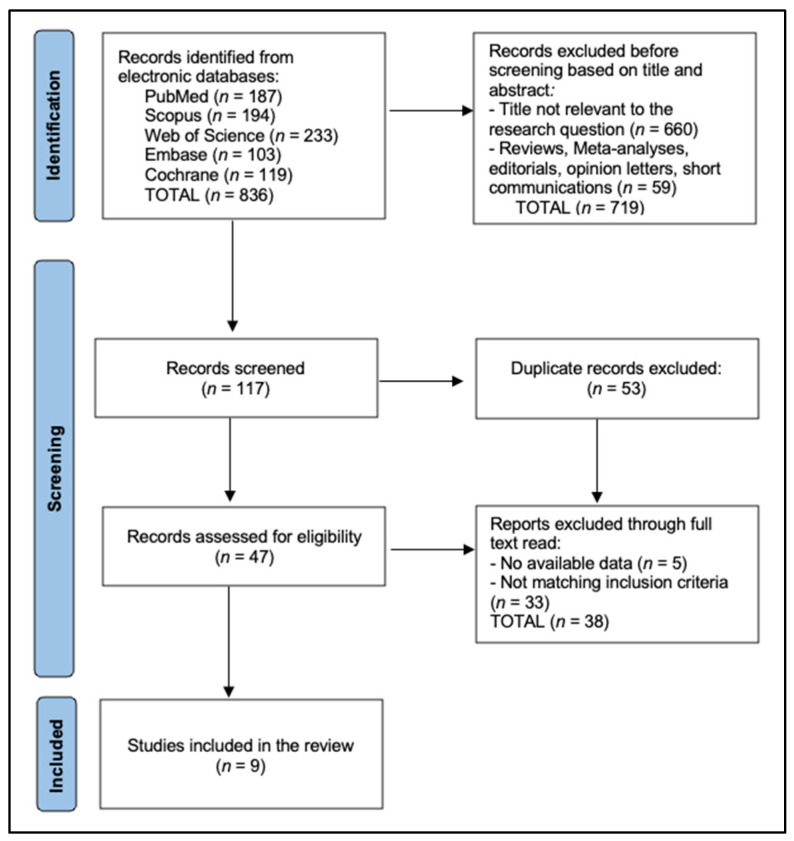
PRISMA 2020 flow diagram.

A total of nine studies were included in the final analysis [23,24,25,26,27,28,29,30,31] (Figure 1), spanning a range of countries, including Brazil, South Korea, and Japan, giving a global context to the research. The studies, such as those by Souza et al. [23], Simoneti et al. [25], and De Castro et al. [29], evaluate materials including SLA resin, bis-acrylic resin, and PMMA, employing fabrication methods like 3D printing, milling, and conventional techniques. Sample sizes in these studies vary significantly, from smaller groups of around 5 samples per test condition in some cases to larger groups of up to 20, which allows for both focused and broader material testing and analysis.

The mechanical properties assessed across these studies are diverse but typically include Vickers and Knoop hardness, flexural strength, elastic moduli, and surface roughness. For example, Souza et al. [23] reported on the Vickers hardness of different resins, finding significant variability; Cosmos Temp (SLA resin) showed much lower hardness at 10.90 VHN compared to Evolux PMMA at 29.11 VHN and Structur 2 SC at 33.37 VHN. Similarly, Simoneti et al. [25] highlighted differences in elastic modulus, where SLA resins showed lower values than conventional acrylic and bis-acryl resins, indicating a potentially weaker structural integrity which might be less suitable for high-load-bearing applications in dentistry.

In addition to mechanical testing, some studies like those of Simoneti et al. [25] and De Castro et al. [29] also explored the effect of print orientation on material properties, revealing that such factors can significantly influence outcomes like flexural strength and hardness. For instance, De Castro et al. [29] found that the orientation could drastically enhance the mechanical properties, with certain orientations achieving much higher hardness values, underscoring the importance of considering build orientation in the 3D printing process to optimize the performance of printed dental prostheses. Collectively, these studies not only underscore the capabilities and limitations of various dental materials and fabrication methods but also highlight the critical role of post-processing and orientation in achieving optimal mechanical properties for clinical applications, as presented in Table 2.

Table 3 presents an array of studies examining the flexural strength of various dental materials, highlighting a diversity of results influenced by the type of material and fabrication method employed. The studies by Souza et al. [23] and Cho and Choi [24] provide a foundational comparison between milled, conventionally cured, and 3D-printed resins. Souza et al. [23] found significant variance within their tested materials, with Evolux PMMA (milled resin) showing the highest flexural strength at 111.76 MPa, significantly outperforming the SLA resin Cosmos Temp, which registered only 56.83 MPa. Similarly, Cho and Choi [24] noted that the DLP resin D3P exhibited the highest strength at 146.37 ± 7.52 MPa, substantially stronger than their conventional resin (CON) at 89.54 ± 6.99 MPa, underscoring the potential of advanced resin formulations and processing techniques to achieve superior mechanical properties.

Simoneti et al. [25] expanded on the diversity of materials tested by including SLS and standard acrylic resins. Their findings highlighted SLS resin as having the highest flexural strength among their test groups at 77.3 ± 3.1 MPa, slightly higher than bis-acryl resin and significantly outperforming SLA resin, which showed the lowest strength at 48.9 ± 1.2 MPa. These results suggest that despite the advancements in 3D printing technologies, certain traditional materials and newer SLS processes might still offer better mechanical resilience.

The study by De Castro et al. [29] further complicates the landscape of material performance by examining the effect of build orientation on flexural strength in 3D-printed resins. Their results show that orientation can have a profound impact, with Cosmos-SLA at a 90° orientation not only improving over time but surpassing the control PMMA CAD/CAM material after one year. Additionally, Park et al. [30] provided data on the performance of various resins under load, with SLA resin showing the highest strength and suggesting that when optimized, SLA fabrication could potentially offer the most durable solutions in prosthetic dentistry.

Table 4 provides detailed insights into the hardness of various dental resins across multiple studies, illustrating the variability in material properties as a result of different fabrication methods. The study by Souza et al. [23] assessed the Vickers hardness of three different types of resins. They found that Structur 2 SC (bis-acrylic resin) and Evolux PMMA (milled resin) displayed relatively high hardness values of 33.37 VHN and 29.11 VHN, respectively, with no significant differences between them (*p* > 0.05). However, Cosmos Temp (SLA resin) exhibited significantly lower hardness at 10.90 VHN, indicating that SLA resins might be less suitable for applications where higher hardness is required.

Simoneti et al. [25] expanded the scope by testing additional materials, including acrylic resin, which showed the highest hardness among their groups at 14.2 ± 2.6 kgf/mm^2^. Their findings suggest that traditional acrylic resin, despite newer technologies, still offers substantial hardness compared to newer resin materials. The SLA resin, in their study, again showed the lowest hardness value at 8.4 ± 0.2 kgf/mm^2^, which was significantly lower than both the acrylic and bis-acryl resins. This consistent finding across studies underscores the potential limitations of SLA resins in terms of hardness compared to both newer and traditional alternatives. Moreover, De Castro et al. [29] evaluated the hardness using the Knoop hardness test, which further demonstrated the disparities among 3D-printed resins. Nanolab (3D-printed resin), with a KHN of 31.8 ± 0.9 in the 0° orientation, was noted for having a higher hardness than the control PMMA CAD/CAM material and other 3D-printed resins. In contrast, Cosmos-SLA had the lowest KHN among the resins tested in the same study, at 8.1 ± 0.3.

Cho and Choi [24] evaluated fracture strength and found that additive manufacturing resins like S3Z (SLA), D3Z, and D3P (both DLP) demonstrated comparable or even superior fracture strength relative to milled (MIL) and conventional (CON) resins, suggesting the potential of 3D printing technologies in producing robust dental materials. Similarly, Simoneti et al. [25] reported on the elastic moduli and surface roughness, indicating that SLA and SLS resins have lower elastic moduli compared to traditional materials like acrylic and bis-acryl resins, with SLA resins also showing significantly lower surface roughness, which could suggest a smoother finish post-polishing.

Further insights from De Castro et al. [29] and Park et al. [30] delve into the accuracy and fracture patterns of 3D-printed resins. De Castro et al. [29] found that orientation dramatically affects the accuracy and mechanical properties of printed resins, with a 90° orientation yielding the best results in terms of accuracy and flexural modulus, emphasizing the importance of print orientation in achieving optimal material characteristics. Park et al. [30] discussed how SLA and DLP resins could handle significant mechanical stress, showing specific fracture patterns that reflect the materials’ inherent strength and behavior under load. Lastly, Sampaio et al. [31] highlighted differences in cement film thickness among different resin types, with SLA 3D-printed resins showing the highest film thicknesses, which could impact the clinical handling and fit of dental restorations (Table 5).

## 4. Discussion

### 4.1. Assessment of Findings and Additional Literature

The results of this systematic review highlight the variability in mechanical properties of SLA 3D-printed materials used for provisional dental restorations. While some studies reported that SLA-printed resins exhibit mechanical properties comparable to milled and conventional materials, others found that these resins have inferior flexural strength, hardness, and accuracy. This inconsistency may be attributed to differences in resin compositions, printing parameters, post-processing protocols, and the specific testing methodologies used across the studies.

In studies where SLA-printed resins demonstrated lower mechanical properties, such as those reported by Souza et al. [23] and Simoneti et al. [25], the flexural strength and hardness were significantly lower than those of milled PMMA and bis-acrylic resins. These findings raise concerns about the suitability of certain SLA-printed materials for provisional restorations that must withstand functional occlusal forces. The lower elastic modulus observed in SLA resins may result in restorations that are more prone to deformation under load.

Conversely, Cho and Choi [24], De Castro et al. [29], and Park et al. [30] observed that SLA-printed resins had flexural and fracture strengths comparable to or exceeding those of conventional and milled resins. In particular, Park et al. [30] reported that SLA-printed three-unit fixed dental prostheses exhibited the highest flexural strength among all groups tested. De Castro et al. [29] found that the build orientation significantly influenced the mechanical properties, with the 90° orientation resulting in higher flexural strength after aging.

Build orientation emerged as a critical factor influencing the mechanical properties and accuracy of SLA-printed restorations. De Castro et al. [29] demonstrated that the 90° orientation resulted in the best overall accuracy and higher flexural strength after one year of aging. This suggests that optimizing build orientation can enhance the performance of SLA-printed provisional restorations.

Surface roughness and marginal adaptation are critical factors influencing plaque accumulation, periodontal health, and the longevity of provisional restorations. Studies included in this review, such as those by Wadhwani et al. [26] and Nagata et al. [27], demonstrated that SLA-printed resins could achieve acceptable surface roughness and marginal fit comparable to conventional methods. These findings support the potential of SLA technology in producing clinically acceptable provisional restorations.

Accuracy and cement film thickness are essential for the proper fit of restorations. Sampaio et al. [31] found that SLA-printed provisional restorations had higher cement film thickness compared to conventional materials, which may affect the adaptation and retention of the restorations. This indicates that SLA-printed restorations may require adjustments or optimization in design parameters to achieve better fit.

Regarding repairability, Albahri et al. [28] reported that while repair materials could bond to SLA resin, the shear bond strength was significantly lower compared to the control, indicating potential limitations in the repairability of SLA-printed provisionals. This suggests that in cases where provisional restorations require repair, SLA-printed materials may not perform as well as traditional materials.

The quantitative analysis of various studies on SLA 3D-printed resins reveals they often exhibit inferior mechanical properties compared to traditional dental materials such as PMMA and bis-acrylic resins. Specifically, SLA resins show a significantly lower average hardness, with Vickers hardness numbers around 12 VHN, compared to approximately 30 VHN for traditional materials. Additionally, while the fracture strength of SLA resins averages around 1000 N, similar to traditional options, the elastic modulus is notably lower, averaging about 500 MPa versus over 850 MPa for materials like acrylic and bis-acryl resins. This variability extends to surface roughness and biofilm formation, with SLA resins sometimes matching traditional materials in surface smoothness but generally posing a higher risk for biofilm accumulation. These findings underscore the necessity for ongoing improvements in SLA resin formulations and 3D printing techniques to better meet clinical demands.

Both Saini et al. [14] and Gad et al. [32] explored the complex factors influencing the flexural strength of 3D-printed resins in dentistry, emphasizing the importance of polymerization and fabrication parameters. Saini et al. [14] highlighted significant variability in flexural strength among different 3D printing technologies and resin materials, identifying substantial differences in performance between types like SLA-3D, DLP, and conventional PMMA, with a notable mean difference in flexural strength and high heterogeneity (I2 = 99%). Similarly, Gad and Fouda [32] identified critical variables such as filler addition, printing orientation, and post-polymerization treatments that significantly affect strength. Both studies underscore the nuanced impact of manufacturing techniques and material choices on the mechanical properties of 3D-printed dental prosthetics, suggesting a need for further research to optimize these factors for enhanced clinical outcomes.

Similarly, Moreira et al. [33] examined the effect of post-polymerization methods and 3D printing systems on resins used in occlusal splints, models, and temporary restorations. They found that LCD printing combined with UV oven post-polymerization yielded the highest flexural strength, achieving σ values that underscore the effectiveness of this method in enhancing mechanical properties. Contrastingly, SLA printing combined with microwave post-polymerization exhibited significant dimensional instability, particularly in occlusal splint resins, where a shrinkage of 40.2% was observed. On the other hand, de Castro et al. [34] focused on the impact of build orientation on the accuracy, flexural modulus, and strength of 3D-printed provisional restorations. Their findings highlighted that a 90° print orientation generally resulted in the best overall accuracy and, after a year of aging, superior flexural strength in certain resins, comparable to traditional milled PMMA. This orientation did not affect microhardness significantly, suggesting that while build orientation can markedly influence dimensional accuracy and strength, its impact on hardness is minimal. These studies collectively emphasize the critical roles that printing technique, post-processing, and orientation play in optimizing the functional properties of 3D-printed dental materials.

Moreover, the findings of the study by Sokola et al. [35] explored the optimization of stereolithography (SLA) and digital light processing (DLP) for printing zirconia-based objects, and the systematic review conducted by Della Bona et al. provided a broad analysis of stereolithography-based 3D printing of restorative materials. Both studies highlighted the need for improving processing factors, such as viscosity and particle size, to ensure high-quality outcomes. Sokola et al. [35] emphasized the prevention of particle agglomeration and sedimentation to align with quality standards, whereas Della Bona et al. [18] focused on the clinical applicability of these materials, indicating that though the technology is promising, there are significant limitations in wear resistance, wet strength, and dimensional accuracy that restrict its clinical use. Moreover, Della Bona et al. [18] noted that only five studies out of those reviewed had progressed to clinical application, suggesting a gap between technological capability and clinical implementation. This mirrors the caution advised by Sokola et al. [35] in fully adopting these technologies without further refinement and validation of the processes involved.

In the context of our systematic review on the quality and mechanical properties of SLA 3D-printed materials for provisional dental restorations, the insights from four recent studies provide a valuable perspective on the varying capabilities and limitations of this technology. Meissner et al. [36] demonstrate that 3D-printed polyamide 12, despite showing higher rigidity and resistance to mechanical and thermal aging than conventionally processed counterparts, also presents challenges such as increased surface roughness and significant color changes, which can affect the aesthetic and functional qualities of dental appliances. Paradowska-Stolarz et al. [37] add to this by illustrating differences in mechanical properties and texture among various 3D-printed dental resins, suggesting that material selection should be strategic based on specific clinical requirements.

Additionally, Wajda et al. [38] highlight the precision of 3D printing in creating indirect bonding transfer trays with accuracy that falls within clinically acceptable ranges, though they note significant variability that could influence orthodontic treatment outcomes. Similarly, Etajuri et al. [39] discuss the use of 3D-printed surgical guides for dental implant placement, emphasizing their potential to achieve clinically acceptable deviations but also pointing out the limitations in achieving absolute precision.

These studies collectively affirm the findings from our review, which observes that while SLA 3D printing technology offers significant promise for fabricating provisional dental restorations with potentially acceptable mechanical properties and customization capabilities, there are evident inconsistencies in material performance. These include variable flexural strength, hardness, and fracture resistance compared to more traditional fabrication methods. The current body of research, including the studies mentioned, indicates a crucial need for further investigation and optimization of SLA materials and printing techniques to harness their full potential and ensure their reliability and effectiveness in clinical dental applications.

The diverse mechanical properties of SLA 3D-printed materials, including variations in flexural strength and hardness, alongside more positive attributes such as comparable fracture strength and acceptable marginal adaptation, indicate that SLA could replace traditional manufacturing methods in certain clinical scenarios. Nonetheless, the challenges posed by increased cement film thickness and the reduced repairability and fatigue resistance of some SLA resins highlight the need for a nuanced understanding of their clinical implications. These limitations underscore the critical gap between the potential of SLA technologies and their practical clinical adoption. To bridge this gap, further research is essential to optimize printing parameters and develop materials that meet the demanding mechanical requirements of provisional dental restorations. This advancement is crucial for the wider acceptance and effectiveness of SLA 3D printing in enhancing patient outcomes.

### 4.2. Study Limitations

This systematic review is limited by the small number of included studies and the heterogeneity among them in terms of materials tested, printing technologies, and evaluation methods. The variations in study designs, specimen geometries, and testing protocols make it challenging to draw definitive conclusions about the overall quality of SLA 3D-printed materials for provisional restorations. Additionally, all included studies are in vitro experiments, which may not fully replicate the complexities of the oral environment. Further clinical studies are necessary to validate the in vitro findings and assess the long-term performance of SLA-printed provisional restorations. The discrepancies among studies highlight the importance of standardizing materials and methods in research and clinical practice. Factors such as layer thickness, printing orientation, post-curing processes, and the specific resin formulations used can significantly influence the mechanical properties of SLA-printed restorations. Establishing guidelines for material properties, printing parameters, and quality control is essential to ensure consistent and reliable outcomes.

## 5. Conclusions

The evidence from the included studies indicates that the mechanical properties and quality of SLA 3D-printed materials for provisional dental restorations vary significantly. While SLA technology offers potential advantages in customization and production efficiency, inconsistencies in material properties highlight the need for careful material selection and optimization of printing parameters. Build orientation, post-processing protocols, and resin composition are critical factors affecting the performance of SLA-printed restorations. Standardization of protocols and further research are essential to ensure that SLA 3D-printed provisional restorations can reliably meet the mechanical demands of clinical use. Clinicians should exercise caution when selecting SLA-printed materials for provisional restorations and consider the specific resin formulations and printing parameters to achieve optimal outcomes.

## Figures and Tables

**Table 1:** Characteristics of included studies.

Database	Search Equation
PubMed	(“stereolithography”[Mesh] OR “stereolithography”[tiab] OR “SLA”[tiab] OR “3D printing”[tiab] OR “additive manufacturing”[tiab]) AND (“provisional dental restorations”[tiab] OR “temporary crowns”[tiab] OR “crowns”[tiab]) AND (“mechanical properties”[tiab] OR “flexural strength”[tiab] OR “hardness”[tiab])
Scopus	TITLE-ABS-KEY(“stereolithography” OR “SLA” OR “3D printing” OR “additive manufacturing”) AND TITLE-ABS-KEY(“provisional dental restorations” OR “temporary crowns” OR “crowns”) AND TITLE-ABS-KEY(“mechanical properties” OR “flexural strength” OR “hardness”)
Web of Science	TS = (“stereolithography” OR “SLA” OR “3D printing” OR “additive manufacturing”) AND TS = (“provisional dental restorations” OR “temporary crowns” OR “crowns”) AND TS = (“mechanical properties” OR “flexural strength” OR “hardness”)
Embase	(’stereolithography’/exp OR stereolithography:ti,ab OR SLA:ti,ab OR ’3D printing’:ti,ab OR ’additive manufacturing’:ti,ab) AND (’provisional dental restorations’:ti,ab OR ’temporary crowns’:ti,ab OR crowns:ti,ab) AND (’mechanical properties’:ti,ab OR ’flexural strength’:ti,ab OR hardness:ti,ab)
Cochrane	(“stereolithography” OR “SLA” OR “3D printing” OR “additive manufacturing”) in Title, Abstract, and Keywords AND (“provisional dental restorations” OR “temporary crowns” OR “crowns”) in Title, Abstract, and Keywords AND (“mechanical properties” OR “flexural strength” OR “hardness”) in Title, Abstract, and Keywords

**Table 2:** Characteristics of included studies.

Study ID	Authors	Year	Country	Study Design	Materials Tested	Fabrication Methods	Sample Size	Mechanical Properties Assessed
1	Souza et al. [23]	2023	Brazil	In vitro	Evolux PMMA (milled resin), Cosmos Temp (SLA resin), Structur 2 SC (bis-acrylic resin, control)	Milling, SLA 3D printing, conventional	n = 12 (flexural strength), n = 9 (hardness)	Flexural strength, Vickers hardness
2	Cho and Choi [24]	2019	South Korea	In vitro	S3Z (SLA resin), D3Z and D3P (DLP resins), MIL (milled resin), CON (conventional self-cured resin)	SLA and DLP 3D printing, milling, conventional	n = 8 (fracture strength), n = 10 (flexural strength)	Fracture strength, flexural strength
3	Simoneti et al. [25]	2020	Brazil	In vitro	Acrylic resin, Bis-acryl resin, SLS resin, SLA resin	Conventional techniques, SLS and SLA 3D printing	n = 10 (flexural strength), n = 5 (hardness), n = 10 (surface roughness), n = 6 (biofilm formation)	Flexural strength, Vickers microhardness, surface roughness, biofilm formation
4	Wadhwani et al. [26]	2022	India	In vitro	SLA resin (Formlabs), DLP resin (Sprintray)	SLA and DLP 3D printing	n = 16 (8 per group)	Surface roughness, marginal adaptation
5	Nagata et al. [27]	2023	Japan	In vitro	PLA models (FDM), Resin models (SLA), Plaster models	FDM 3D printing, SLA 3D printing, Conventional casting	n = 5 per group	Marginal fit
6	Albahri et al. [28]	2021	USA/South Korea	In vitro	SLA resin repaired with PMMA, Bis-acrylic composite, Bis-GMA composite	SLA 3D printing, repair materials application	n = 15 per group	Shear bond strength
7	De Castro et al. [29]	2022	Brazil	In vitro	PMMA CAD/CAM (Control), Cosmos-SLA, Cosmos-DLP, PriZma-Bioprov, Nanolab	Milling (control), 3D printing (SLA, DLP)	n = 20 (bars), n = 10 (discs)	Accuracy, flexural modulus, flexural strength, microhardness
8	Park et al. [30]	2020	South Korea	In vitro	CV (Self-curing PMMA), SM (milled PMMA), DLP resin, SLA resin, FDM resin (PLA)	Conventional, milling, 3D printing (DLP, SLA, FDM)	n = 15 per group	Flexural strength
9	Sampaio et al. [31]	2020	Chile	In vitro	Acrylic resin, Bis-acrylic resin, PMMA CAD/CAM, 3D-printed resin	Conventional, CAD/CAM, 3D printing (SLA)	n = 6 per group (veneers), n = 6 per group (crowns)	Cement film thickness (via micro-CT)

PMMA—polymethylmethacrylate; SLA—stereolithography; DLP—digital light processing; FDM—fused deposition modeling; SLS—selective laser sintering; CAD/CAM—computer-aided design/computer-aided manufacturing; micro-CT—micro-computed tomography; Bis-GMA—bisphenol A-glycidyl methacrylate; PLA—polylactic acid.

**Table 3:** Flexural strength results.

Study ID	Authors	Materials Tested	Flexural Strength (Mean ± SD or Median, Units), MPa	Statistical Findings
1	Souza et al. [23]	Evolux PMMA (milled resin)	111.76	Significantly higher than Cosmos Temp and Structur 2 SC (*p* < 0.05)
		Structur 2 SC (bis-acrylic resin)	87.34	Intermediate value
		Cosmos Temp (SLA resin)	56.83	Significantly lower than Evolux PMMA and Structur 2 SC (*p* < 0.05)
2	Cho and Choi [24]	S3Z (SLA resin)	116.08 ± 14.46	Significantly higher than CON (*p* < 0.05)
		D3Z (DLP resin)	46.83 ± 3.44	Significantly lower than CON (*p* < 0.05)
		D3P (DLP resin)	146.37 ± 7.52	Significantly higher than CON (*p* < 0.05)
		MIL (milled resin)	168.57 ± 2.06	Highest among all groups
		CON (conventional self-cured resin)	89.54 ± 6.99	Reference group
3	Simoneti et al. [25]	Acrylic resin	69.2 ± 8.8	Intermediate value
		Bis-acryl resin	75.0 ± 8.2	Intermediate value
		SLA resin	48.9 ± 1.2	Lowest among groups (significantly lower)
		SLS resin	77.3 ± 3.1	Highest among groups in this study
7	De Castro et al. [29]	Cosmos-SLA (3D-printed resin)	FS after 24h: 96.4 ± 6.5 (0°), 103.9 ± 3.2 (45°), 109.7 ± 5.1 (90°)	After 1 year, FS of 90°, Cosmos-SLA (120.5 ± 4.3 MPa) higher than Control; build orientation influenced FS
		Cosmos-DLP (3D-printed resin)	FS decreased after 1 year; e.g., 88.4 ± 3.9 (0°) after 24 h to 73.1 ± 2.7 after 1 year	Build orientation influenced FS
		PriZma-Bioprov (3D-printed resin)	FS after 1 year similar to Control; e.g., 102.3 ± 5.7 (90°) after 1 year	After 1 year, FS similar to control
		Nanolab (3D-printed resin)	FS decreased after 1 year; e.g., 84.5 ± 4.0 (0°) after 24 h to 77.2 ± 3.5 after 1 year	FS lower than control
		Control (PMMA CAD/CAM material)	FS after 24 h: 131.9 ± 4.8; After 1 year: 126.7 ± 3.9	Control had highest FS at both time points
8	Park et al. [30]	CV (conventional self-curing PMMA)	Median 543 N (IQR 429–701 N)	Significantly lower than DLP and SLA groups (*p* < 0.001)
		SM (milled PMMA)	Median 1232 N (IQR 1193–1258 N)	No significant difference compared to DLP (*p* = 0.481)
		DLP (3D-printed resin)	Median 1189 N (IQR 1110–1283 N)	No significant difference compared to SM (*p* = 0.481)
		SLA (3D-printed resin)	Median 1323 N (IQR 1245–1377 N)	Significantly higher than other groups (*p* < 0.001)
		FDM (3D-printed resin)	No fracture observed (specimens only dented)	Not included in statistical analysis

PMMA—polymethylmethacrylate; SLA—stereolithography; DLP—digital light processing; FDM—fused deposition modeling; SLS—selective laser sintering; CAD/CAM—computer-aided design/computer-aided manufacturing; MPa—megapascal; FS—flexural strength; SD—standard deviation; IQR—interquartile range.

**Table 4:** Hardness results.

Study ID	Authors	Materials Tested	Vickers Hardness (Mean ± SD, VHN)	Statistical Findings
1	Souza et al. [23]	Structur 2 SC (bis-acrylic resin)	33.37 VHN	No significant difference compared to Evolux PMMA (*p* > 0.05)
		Evolux PMMA (milled resin)	29.11 VHN	No significant difference compared to Structur 2 SC (*p* > 0.05)
		Cosmos Temp (SLA resin)	10.90 VHN	Significantly lower than both Structur 2 SC and Evolux PMMA (*p* < 0.05)
3	Simoneti et al. [25]	Acrylic resin	14.2 ± 2.6 kgf/mm^2^	Highest hardness among groups
		Bis-acryl resin	10.7 ± 2.2 kgf/mm^2^	Intermediate value
		SLA resin	8.4 ± 0.2 kgf/mm^2^	Lowest among groups (significantly lower)
		SLS resin	10.3 ± 1.0 kgf/mm^2^	Intermediate value
7	De Castro et al. [29]	Nanolab (3D-printed resin)	31.8 ± 0.9 KHN (0° orientation)	Higher KHN than Control and other 3DRs
		PriZma-Bioprov (3D-printed resin)	25.2 ± 0.5 KHN (0° orientation)	Higher KHN than Cosmos-DLP and Cosmos-SLA
		Cosmos-DLP (3D-printed resin)	9.8 ± 0.4 KHN (0° orientation)	Lower KHN than Control and PriZma
		Cosmos-SLA (3D-printed resin)	8.1 ± 0.3 KHN (0° orientation)	Lowest KHN among 3DRs
		Control (PMMA CAD/CAM material)	23.1 ± 0.7 KHN	Lower KHN than Nanolab, higher than Cosmos-SLA and Cosmos-DLP

VHN—Vickers hardness number; KHN—Knoop hardness number; PMMA—polymethylmethacrylate; SLA—stereolithography; DLP—digital light processing; SLS—selective laser sintering; CAD/CAM—computer-aided design/computer-aided manufacturing; kgf/mm^2^—kilogram-force per square millimeter; 3DR—3D resin; SD—standard deviation.

**Table 5:** Other mechanical properties and findings.

Study ID	Author	Properties Assessed	Results	Key Findings
2	Cho and Choi [24]	Fracture strength	S3Z (SLA resin): 987.50 ± 74.37 N; D3Z: 1020.99 ± 139.13 N; D3P: 1069.15 ± 153.23 N; MIL: 748.49 ± 135.61 N; CON: 678.48 ± 152.16 N	Additive manufacturing resins had comparable or higher fracture strength compared to milling and conventional methods
3	Simoneti et al. [25]	Elastic moduli	SLA resin: 513.3 ± 29.7 MPa; SLS resin: 452.4 ± 35.8 MPa; acrylic resin: 859.4 ± 46.3 MPa; bis-acryl resin: 997.3 ± 108.5 MPa	SLA and SLS resins had lower elastic moduli compared to conventional materials
		Surface roughness (after polishing)	SLA resin: 0.7 ± 0.1 μm; bis-acryl resin: 0.7 ± 0.1 μm; acrylic resin: 0.9 ± 0.2 μm; SLS resin: 1.2 ± 0.3 μm	SLA resin had the lowest surface roughness, comparable to bis-acryl resin
		Biofilm formation	Biofilm mass (OD570): SLA resin: 0.28 ± 0.03; SLS resin: 0.27 ± 0.03; acrylic resin: 0.29 ± 0.02; bis-acryl resin: 0.28 ± 0.03	No significant differences among materials (*p* = 0.949)
4	Wadhwani et al. [26]	Surface roughness	SLA resin: Ra = 1.236 ± 0.136 μm; DLP resin: Ra = 1.932 ± 0.258 μm	SLA group had significantly lower surface roughness than DLP group (*p* < 0.05)
		Marginal adaptation	SLA resin: marginal gap = 54.60 ± 14.22 μm; DLP resin: marginal gap = 84.58 ± 16.42 μm	SLA group showed significantly better marginal adaptation compared to DLP group (*p* < 0.005)
5	Nagata et al. [27]	Marginal fit	SLA models: mean gap = 50.4 ± 7.2 μm; plaster models: mean gap = 47.6 ± 6.8 μm; FDM models: mean gap = 68.9 ± 9.1 μm	No significant difference between SLA and plaster models except at one point; FDM models had larger gaps
6	Albahri et al. [28]	Shear bond strength	Group A (control): 253 ± 10 N; Group B (PMMA): 167 ± 29 N; Group C (bis-acryl composite): 174 ± 25 N; Group D (bis-GMA composite): 196 ± 37 N	Repaired SLA specimens had significantly lower shear bond strength than control (*p* < 0.001)
7	De Castro et al. [29]	Accuracy	Length percent error: Cosmos-SLA (90°): 0.02%; thickness percent error: Cosmos-SLA (90°): 0.12%; width percent error: Cosmos-SLA (90°): 0.08%	90° orientation resulted in best overall accuracy for most 3DRs; Cosmos-SLA accuracy comparable to milled PMMA
		Flexural modulus (FM)	Cosmos-SLA: FM after 24 h: 2.1 ± 0.1 GPa; after 1 year: 2.3 ± 0.1 GPa	FM of all 3DRs was lower than control (Control FM after 24 h: 3.6 ± 0.1 GPa)
		SEM and EDS Analyses	Nanolab resin had irregular filler particles (~2–14 μm); Cosmos-SLA had few nanometric spherical particles	Nanolab presented irregular fillers affecting properties
8	Park et al. [30]	Fracture patterns	CV group: fractures occurred at connector area; SLA group: fractures at pontic area with small fragments; DLP group: fractures into several pieces	DLP and SLA materials provided adequate flexural strength; FDM specimens did not fracture but dented
		Surface evaluation (FESEM)	DLP specimens showed stepped layers; SLA specimens had well-filled layers; FDM specimens had visible layer lines	Manufacturing method affects SLA surface characteristics
9	Sampaio et al. [31]	Cement film thickness	Veneers: bis-acrylic resin: 0.09 ± 0.04 mm; acrylic resin: 0.14 ± 0.03 mm; PMMA CAD/CAM: 0.18 ± 0.03 mm; 3D-printed resin: 0.32 ± 0.03 mm	SLA 3D-printed resin showed highest cement film thickness; veneers had smaller thickness than crowns
			Crowns: PMMA CAD/CAM: 0.24 ± 0.04 mm; bis-acrylic resin: 0.28 ± 0.08 mm; acrylic resin: 0.34 ± 0.06 mm; 3D-printed resin: 0.62 ± 0.08 mm	SLA 3D-printed resin had significantly higher cement film thickness compared to other materials

N—Newton; MPa—megapascal; GPa—gigapascal; μm—micrometer; Ra—roughness average; OD570—optical density at 570 nm; SEM—scanning electron microscopy; EDS—energy-dispersive X-ray spectroscopy; FESEM—field emission scanning electron microscopy; SLA—stereolithography; DLP—digital light processing; FDM—fused deposition modeling; SLS—selective laser sintering; PMMA—polymethylmethacrylate; 3DR—3D resin; kgf/mm^2^—kilogram-force per square millimeter; VHN—Vickers hardness number; KHN—Knoop hardness number.

## Data Availability

No new data were created or analyzed in this study.
